# Early Diagnosis and Treatment of Cardiac Amyloidosis by Screening Biopsy During Trigger Finger Release

**DOI:** 10.1016/j.jhsg.2024.07.013

**Published:** 2024-08-29

**Authors:** Ella Gibson, Imo A. Ebong, Morgan A. Darrow, Ge Xiong, Angelo B. Lipira, Ravi F. Sood

**Affiliations:** ∗ Division of Plastic and Reconstructive Surgery, Department of Surgery, University of California Davis, Sacramento, CA; † Division of Cardiology, Department of Internal Medicine, University of California Davis, Sacramento, CA; ‡ Department of Pathology and Laboratory Medicine, University of California Davis, Sacramento, CA; § Department of Neurology, University of California Davis, Sacramento, CA; ‖ Division of Plastic and Reconstructive Surgery, Department of Surgery, Oregon Health & Science University, Portland, OR

**Keywords:** Amyloid, Amyloidosis, Amyloidosis screening, Cardiac amyloidosis, Stenosing flexor tenosynovitis, Trigger digit, Trigger finger

## Abstract

Patients undergoing trigger release surgery are known to be at increased risk of amyloidosis and heart failure, and therefore, amyloidosis screening during trigger release surgery may facilitate early diagnosis and treatment of cardiac amyloidosis. However, the reported prevalence of amyloid on biopsies taken during trigger release surgery has varied widely, and no biopsy-positive patients in prior studies have been diagnosed with occult cardiac amyloidosis or started on disease-modifying therapy. We review the existing literature on this topic and present a case of a patient with cardiac amyloidosis diagnosed from a biopsy taken during trigger release surgery and subsequently started on disease-modifying therapy. This case supports the potential role of amyloidosis screening during trigger release and highlights the benefits of collaboration between hand surgeons and amyloidosis specialists in multidisciplinary amyloidosis programs.

The amyloidoses are a heterogeneous group of disorders caused by the extracellular deposition of insoluble amyloid fibrils into various tissues including the heart, kidneys, liver, nerves, and gastrointestinal and musculoskeletal systems. Amyloid fibrils disrupt the architecture of the affected organ(s) to cause clinical manifestations of the disease. Cardiac involvement in patients with systemic amyloidosis is the strongest predictor of mortality and is common in two of the most prevalent types, transthyretin (TTR) amyloidosis and immunoglobulin light chain amyloidosis. The development of heart failure carries an extremely poor prognosis, with median survival rates of 6 months and 4 years after presentation in patients with immunoglobulin light chain amyloidosis and TTR amyloidosis, respectively.^1^ Although treatment of cardiac amyloidosis was previously limited to symptom management, disease-modifying therapies are now available. The mechanisms of action of these drugs focus on preventing disease progression by either stabilizing precursor proteins or inhibiting the synthesis of these proteins. Because therapy for amyloid cardiomyopathy is most effective when initiated before the onset of clinically apparent cardiac dysfunction, early identification of affected individuals is critical to prevent morbidity and mortality related to heart failure or conduction abnormalities.^2^

Musculoskeletal conditions such as carpal tunnel syndrome, biceps tendon rupture, spinal stenosis, and rotator cuff disease are also frequently seen in amyloidosis, and these manifestations often precede cardiac disease by several years.^3^ Accordingly, there has been interest in amyloidosis screening during routine orthopedic procedures. For instance, screening tenosynovial biopsy at the time of carpal tunnel release is increasingly performed for patients at increased risk of amyloidosis, although the optimal criteria for screening have yet to be defined.^1^^,^^4^

Trigger digit, or stenosing flexor tenosynovitis, is another common musculoskeletal condition associated with amyloidosis, estimated to affect 2% of the general adult population. There is an increased incidence of amyloidosis and heart failure among patients undergoing trigger release surgery; therefore, amyloidosis screening during trigger release surgery represents another potential means of early diagnosis and treatment of amyloidosis.^5^ However, the few studies available on this topic report the widely variable prevalence of amyloid among biopsies performed during trigger release (2% to 65%), and no biopsy-positive patients in published studies have been diagnosed with occult cardiac amyloidosis or started on disease-modifying therapy.^6^, ^7^, ^8^, ^9^, ^10^ We present a case of a patient with cardiac amyloidosis diagnosed from a biopsy taken during trigger release surgery and subsequently started on disease-modifying therapy. We also review the existing literature on this topic.

## Case Presentation

Written informed consent was obtained from the patient for publication of this case report and accompanying images. A 71-year-old right hand–dominant white male engineer with a medical history of immunoglobulin G kappa monoclonal gammopathy of unclear significance (MGUS), longstanding peripheral neuropathy, and multiple prior trigger digit releases developed left index finger triggering of about 1 year’s duration for which he was referred to hand surgery. Given the patient’s peripheral neuropathy of uncertain etiology and clinical suspicion of amyloidosis being evaluated by his neurologist, the patient had undergone an ultrasound-guided abdominal fat pad biopsy 2 months prior to his presentation for trigger finger, which returned negative. Considering his age, history of multiple trigger digits, MGUS, and peripheral neuropathy, we recommended a biopsy at the time of his trigger release surgery to evaluate for amyloidosis.

**Figure 1:**
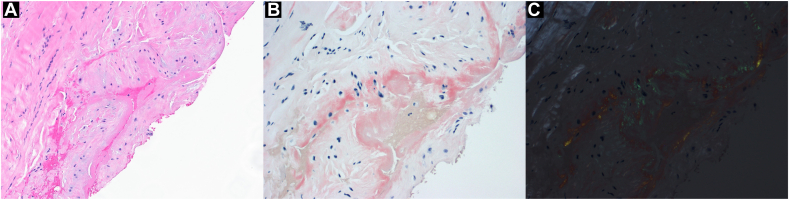
Hematoxylin-eosin stain of A1 pulley biopsy taken during trigger release surgery (**A**, magnification × 200) and Congo red stain (magnification × 400) without (**B**) and with (**C**) polarized light, the latter demonstrating characteristic apple-green birefringence.

A standard A1 pulley release was performed under a local anesthetic. Intraoperative findings included a thick nodule of the flexor tendons at the level of the A1 pulley along with thickening of the pulley. As there was no tenosynovium evident within the tendon sheath at this level, a full-thickness portion of the A1 pulley measuring approximately 5 × 5 mm (corresponding to about half the proximal-to-distal length of the pulley) was sent to pathology. Congo red staining demonstrated foci of amyloid deposition (Fig. 1), and subtyping by mass spectrometry was consistent with TTR amyloidosis. Genetic testing was negative for transthyretin mutation, confirming wild-type transthyretin amyloidosis.

**Figure 2:**
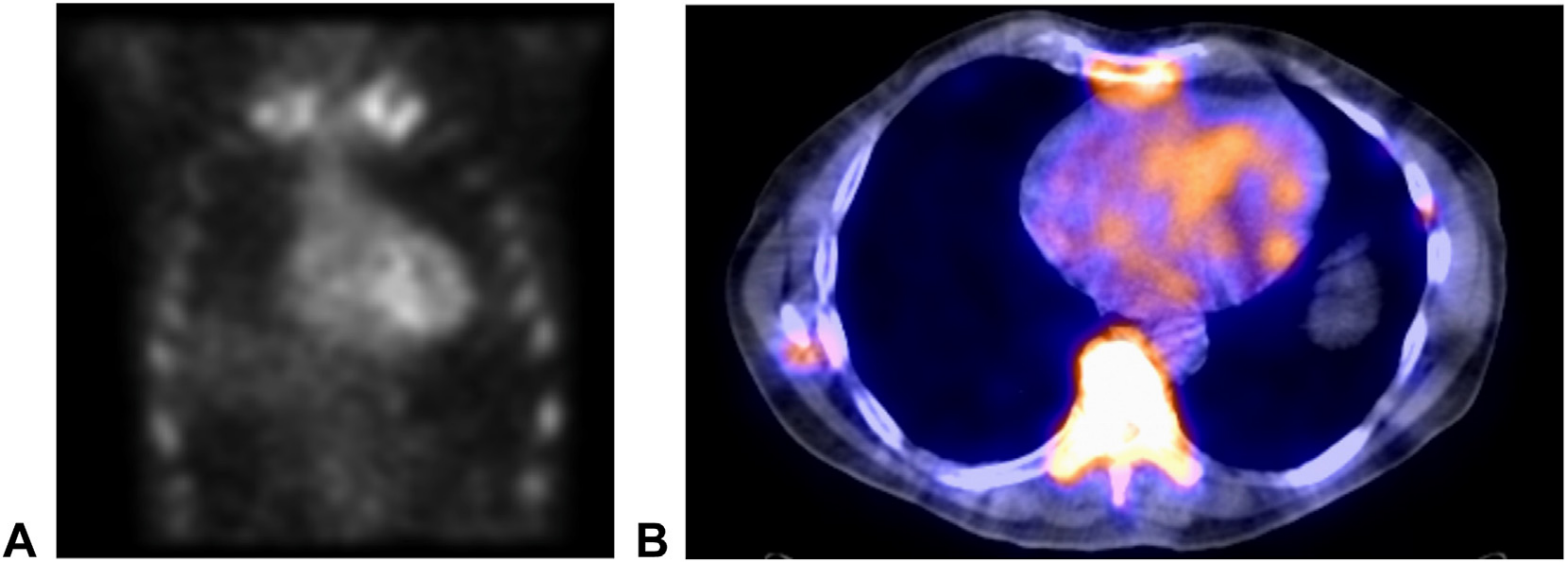
Technetium-99m pyrophosphate scan **A** with SPECT-CT (single-photon emission computed tomography [SPECT] combined with low-dose non-contrast computed tomography [CT]) **B** demonstrating increased radiotracer uptake involving all four walls of the left ventricle, supporting the diagnosis of cardiac amyloidosis.

The patient recovered uneventfully from his operation with resolution of his trigger finger and was referred to the cardiac amyloidosis clinic for further evaluation. He had good functional status without chest pain, dyspnea on exertion, orthopnea, paroxysmal nocturnal dyspnea, lower-extremity edema, or syncope. Echocardiogram obtained 1.5 months after surgery revealed preserved left ventricular function (60%), moderate right ventricular dilation in the setting of normal pulmonary artery pressures, and abnormal left ventricular global longitudinal strain of −10%. At 5.5 months after surgery, the technetium-99m pyrophosphate scan revealed increased radiotracer uptake involving all four walls of the left ventricle (Fig. 2), and cardiac magnetic resonance imaging demonstrated diffuse T1 signal uptake within the left ventricular myocardium, supporting the diagnosis of cardiac amyloidosis. The patient was started on tafamidis and remains asymptomatic from a cardiac standpoint 3 months after diagnosis and initiation of therapy for cardiac amyloidosis.

## Discussion

With the recent advent of effective therapeutics to prevent the progression of amyloidosis, there is a growing public health effort toward developing screening systems for this deadly group of diseases that until recently was considered untreatable. As musculoskeletal manifestations often precede cardiac involvement in systemic amyloidosis, hand surgeons can facilitate the detection of systemic disease at earlier stages to allow for earlier initiation of potentially life-saving treatment. This paradigm is becoming well established in the surgical treatment of carpal tunnel syndrome, with the prevalence of amyloid among biopsies taken during carpal tunnel release estimated to be between 10% and 29%, and with corresponding reports of disease-modifying therapy for cardiac amyloidosis being initiated from screening in this manner.^3^ However, few data exist regarding screening for amyloid at the time of trigger finger release (Table).^6^, ^7^, ^8^, ^9^, ^10^

**Table:** Summary of Published Studies of Amyloidosis Screening During Trigger Digit Release

Year	Lead Author	City	Design	Inclusion Criteria	Exclusion Criteria	Patients (N)	Mean Age (y)	Gender	Biopsy Technique	Positive Biopsy			Cardiac Amyloidosis		
										N	%	Amyloid Type	Diagnostic Work-up	Cases Diagnosed	Treatment Started
2001	Cordiner-Lawrie et al^6^	Oxford, UK	NR	TD refractory to corticosteroid injection	Diabetes	47	45	31 (66%) women, 16 (34%) men	Tendon sheath, congo red staining, typing by IHC	11	23	Undetermined∗	NR	NR	NR
2020	Çatal et al^7^	Istanbul, Turkey	Prospective cohort	Idiopathic TD	CKD on HD, congenital TD, gout, known amyloidosis, pregnancy, prior corticosteroid injection, RA, tuberculous synovitis	68	58	50 (74%) women, 18 (26%) men	Tenosynovium, congo red staining, typing by IHC	6	9	6/6 (100%) TTR	ECG, TTE	0	N/A
2020	Hara et al^8^	Tokyo, Japan	Retrospective cohort	NR	Diabetes, CKD on HD, RA	20	65	13 (65%) women, 7 (35%) men	Tenosynovium, direct fast scarlet staining, typing by IHC	13	65	12/13 (92%) TTR†	ECG, TTE	0	N/A
2021	Sperry et al^9^	Cleveland, OH	Prospective cohort	Age ≥ 50 y	None	100	66	59 (59%) women, 41 (41%) men	Tenosynovium, congo red staining, typing by mass spectrometry	2	2	2/2 (100%) TTR	ECG, TTE, t-99m pyrophosphate nuclear scintigraphy	0	N/A
2023	Gray et al^10^	Indianapolis, IN	Prospective cohort	Men ≥ 50 y or women ≥ 60 y and multiple TDs or TD(s) and CTS	None	17	NR	9 (53%) women, 8 (47%) men	Tenosynovium, congo red staining	4	24	NR	NR	NR	NR

CKD, chronic kidney disease; CTS, carpal tunnel syndrome; ECG, electrocardiogram; HD, hemodialysis; IHC, immunohistochemistry; N/A, not applicable; NR, not reported; RA, rheumatoid arthritis; TD, trigger digit; TTE, transthoracic echocardiogram; TTR, transthyretin.

∗ Negative for amyloid A, β-2-microglubulin, immunoglobulin light chain, and TTR by immunohistochemistry.

† Nine specimens showed extensive (>50%) staining for TTR, and three specimens showed partial (25% to 50%) staining.

Çatal et al^7^ reported a 9% prevalence of tenosynovial amyloid among samples taken during trigger release in a cohort of 68 consecutive adults at two centers in Turkey. All six amyloid-positive patients had transthyretin type and negative workups for cardiac amyloidosis. In a smaller cohort study from Japan, Hara et al^8^ found that 13 of 20 (65%) screened adults had amyloid detected on biopsies, the majority (12/13, 92%) transthyretin type, all of whom had negative evaluations for cardiac amyloidosis. In a prospective cohort of 100 adults over the age of 50 undergoing trigger release at the Cleveland Clinic—the largest study to date—only two (2%) demonstrated amyloid in tenosynovial biopsies, both of which had negative workups for cardiac amyloidosis.^9^ Notably, 13 patients in the cohort underwent carpal tunnel release surgery concomitant with trigger release and had tenosynovial biopsies taken from both surgical sites; of these, two had amyloid detected from tenosynovium in the carpal tunnel with negative biopsies from the fingers. Gray et al^10^ screened nine men over the age of 50 years and eight women over the age of 60 years presenting with multiple trigger digits and found amyloid in four (24%) of tenosynovial specimens; however, cardiac evaluation was not reported.

Due to limitations in these studies, the role of screening for amyloidosis during trigger release has yet to be defined. The wide variation in amyloid detection (ranging: 2% to 65%) likely reflects differences in the patient populations and the biopsy technique/tissue sampled. The Japanese study reports by far the highest prevalence of amyloid among biopsies from trigger release surgery (65%), but inclusion criteria for biopsy are not reported, and it may be that higher-risk patients were screened.^8^ Moreover, Japanese studies of screening biopsies during carpal tunnel release have also shown consistently higher positivity rates than those from other countries, suggesting a possible genetic influence. In contrast, the largest study, to date, reported only a 2% prevalence of amyloid among biopsies during trigger release.^9^ However, the only inclusion criterion for biopsy was age ≥50, and their cohort was predominantly women. The diagnostic yield might, therefore, have been much higher if more stringent criteria for screening were applied (ie, more clinical risk factors for amyloidosis required for biopsy), as has been performed in screening during carpal tunnel release. In addition, biopsies were limited to tenosynovium, which is often scarce or absent in the region of the A1 pulley. Indeed, the authors conclude that sampling error may have led to an unexpectedly low positivity rate. It may be that sampling the A1 pulley itself, as we did in the reported case for precisely this reason, could mitigate the issue of false-negative biopsies because of sampling error. That our patient had a negative abdominal fat pad biopsy prior to his trigger release supports this notion, as do the findings of Cordiner-Lawrie et al,^6^ who reported a 23% prevalence of amyloid among tendon sheath biopsies from 46 adults in the United Kingdom. Finally, although none of these studies reported cardiac amyloidosis diagnosed at the time of biopsy, the patients were not followed serially, and patients with amyloid deposition detected during trigger release or carpal tunnel may develop cardiac amyloidosis during follow-up after an initially negative cardiac work-up. The incidence of cardiac amyloidosis in such patients with positive biopsies for amyloid during carpal tunnel release surgery is similarly unknown.^3^

This case along with epidemiologic evidence demonstrating elevated risk of amyloidosis and heart failure among patients undergoing trigger release supports the potential role of amyloidosis screening during this common procedure. Existing data suggest that older age, male sex, Black/African American race, history of carpal tunnel syndrome, and multiple trigger digits (three of which were present in our patient) are risk factors to be investigated as potential criteria for screening. The presented case also suggests MGUS as an additional candidate screening criterion, consistent with recent evidence of a higher prevalence of MGUS among not only patients with immunoglobulin light chain amyloidosis but also those with TTR amyloidosis.^11^ However, further studies are required to establish the optimal technique and criteria for screening as well as its cost-effectiveness prior to routine implementation of amyloidosis screening during trigger release. Therefore, at our institution, biopsy for amyloid during trigger release is currently limited to patients actively undergoing evaluation for suspected amyloidosis (in contrast to screening biopsy during carpal tunnel release, which we perform according to a previously published nomogram).^4^ This case also illustrates the benefit of multidisciplinary programs for collaboration between hand surgeons and other specialists with expertise in amyloidosis including cardiologists, neurologists, and hematologists/oncologists.

## Conflicts of Interest

No benefits in any form have been received or will be received related directly to this article.
